# A role for trypanosomatid aldo-keto reductases in methylglyoxal, prostaglandin and isoprostane metabolism

**DOI:** 10.1042/BCJ20180232

**Published:** 2018-08-29

**Authors:** Adam J. Roberts, Joanne Dunne, Paul Scullion, Suzanne Norval, Alan H. Fairlamb

**Affiliations:** Division of Biological Chemistry and Drug Discovery, School of Life Sciences, University of Dundee, Dundee DD1 5EH, U.K.

**Keywords:** aldo-keto reductases, drug metabolism, isoprostane, lipid mediators, methylglyoxal, trypanosomes

## Abstract

Trypanosomatid parasites are the infectious agents causing Chagas disease, visceral and cutaneous leishmaniasis and human African trypanosomiasis. Recent work of others has implicated an aldo-keto reductase (AKR) in the susceptibility and resistance of *Trypanosoma cruzi* to benznidazole, a drug used to treat Chagas disease. Here, we show that *Tc*AKR and homologues in the related parasites *Trypanosoma brucei* and *Leishmania donovani* do not reductively activate monocyclic (benznidazole, nifurtimox and fexinidazole) or bicyclic nitro-drugs such as PA-824. Rather, these enzymes metabolise a variety of toxic ketoaldehydes, such as glyoxal and methylglyoxal, suggesting a role in cellular defence against chemical stress. UPLC-QToF/MS analysis of benznidazole bioactivation by *T. cruzi* cell lysates confirms previous reports identifying numerous drug metabolites, including a dihydro-dihydroxy intermediate that can dissociate to form *N*-benzyl-2-guanidinoacetamide and glyoxal, a toxic DNA-glycating and cross-linking agent. Thus, we propose that *Tc*AKR contributes to benznidazole resistance by the removal of toxic glyoxal. In addition, three of the four enzymes studied here display activity as prostaglandin F_2α_ synthases, despite the fact that there are no credible cyclooxygenases in these parasites to account for formation of the precursor PGH_2_ from arachidonic acid. Our studies suggest that arachidonic acid is first converted non-enzymatically in parasite lysates to (PGH_2_-like) regioisomers by free radical-mediated peroxidation and that AKRs convert these lipid peroxides into isoprostanes, including prostaglandin F_2α_ and 8-iso-prostaglandin F_2α_.

## Introduction

Parasitic protozoa belonging to the order Kinetoplastida cause three neglected tropical diseases, Chagas disease, human African trypanosomiasis and leishmaniasis due to infection with *Trypanosoma cruzi*, *Trypanosoma brucei* and *Leishmania* spp., respectively, see review [1]. The burden of these three ‘neglected’ tropical diseases is almost exclusively associated with poverty and therefore there has been little incentive for pharmaceutical companies to invest in developing new treatments, see review [2]. Currently, the nitro-drugs benznidazole and nifurtimox developed over 40 years ago are the only therapies available for the treatment of Chagas disease, with nifurtimox also constituting part of the combination therapy ‘NECT’ for late-stage human African trypanosomiasis [3]. In addition, another nitro-drug, fexinidazole, has shown activity against all three parasites [4–6] and consequentially is now under clinical assessment for all three diseases.

The anti-parasitic activities of these nitro-containing pro-drugs are dependent upon parasite-facilitated bio-activation by a bacterial-like type I nitroreductase NTR1 [7] to form toxic reactive intermediates [8–10], and consequently, loss of NTR1 is a key resistance determinant [11,12]. Notably, other enzymes have been implicated in resistance to benznidazole in *T. cruzi*, the FMN containing ‘old yellow enzyme’ a functional prostaglandin F_2α_ synthase [13,14] and an aldo-keto reductase (AKR) [15]. Somewhat paradoxically, the latter enzyme has been reported to activate benznidazole, yet overexpression of AKR in *T. cruzi* decreased rather than increased susceptibility to benznidazole [16]. Members of this AKR superfamily, characterised by their ability to reduce aldehydes and/or ketones, have been identified in all domains of life [17]. The activity of these enzymes requires either NADH and/or NADPH to reduce a variety of substrates, including, but not limited to aldoses, steroids, methylglyoxal [18] or prostaglandins [19]. In the trypanosomatid parasites *Leishmania* spp. and *T. brucei*, the production of prostaglandin F_2α_ has been reported to be catalysed by a member of the AKR family [19,20]; however, the equivalent enzyme in *T. cruzi* is reported not to catalyse this reaction [21]. Given the conflicting and contradictory reports in the literature, we have re-examined the ability of TcAKR to metabolise benznidazole and carried out a comparative enzymological study of trypanosomatid AKRs to elucidate their potential metabolic roles in prostanoid and glyoxal metabolism.

## Experimental procedures

### Cloning and expression of recombinant proteins

The open reading frames (ORFs) encoding *LiPGFS1* (LinJ.32.0470, http://tritrypdb.org) and *Hs*PGFS (NP_003730.4) were synthesised by GeneART^™^. The ORFs of *TcAKR* (GenBank MH593394), *TbPGFS* (Tb927.11.4700) and *LiPGF2* (LinJ.31.2210) were amplified from genomic DNA isolated from *T. cruzi* Silvio X10/7 clone A1, *T. brucei* 427 and *Leishmania infantum* genomic DNA, respectively, using primers (Table 1) and pfu polymerase. The resulting PCR products were cloned into TOPO zero blunt. The ORFs of each gene were digested with the appropriate restriction endonucleases and ligated into pET15b-TEV, yielding pET15b-TEV-*HsPGFS,* pET15b-TEV-*TcAKR*, pET15b-TEV-*LiPGFS*1, pET15b-TEV-*LiPGFS*2 and pET15b-TEV-*LiAKR*. All plasmids were sequenced (www.dnaseq.co.uk) to verify the correct sequences.

**Table 1:** List of primers used in the present study

Primer	Sequence
*Tc*AKR-F	catATGAATTGCAATTACAACTGTGTGACACTCC
*Tc*AKR-R	ggatccTCACTCCTCTCCACCAGGG
*Tb*PGFS-F	catATGGCTCTCACTCAATCCCTAAAACTC
*Tb*PGFS-R	ggatccTCAAAAGTCGTTCATGAAGACCTCC
*Li*PGFS2-F	aaaacatATGGCTGACGTTGGTAAGGCAATGG
*Li*PGFS2-R	ttttggatccTTAGAACTGCGCCTCATCGGGGTC
*Tc*PGF2α-F	catATGGCGACGTTCCCCGAAC
*Tc*PGF2α-R	ggatccTTAGTTGTTGTACGTCGGGTAATCG

Restriction sites are underlined.

Plasmids were transformed into Rosetta (DE3) pLysS and 10-ml cultures were used to inoculate 1 l of Autoinduction medium [22] with shaking at 30°C for 12 h. Cells were harvested by centrifugation (5090×***g*** for 30 min at 4°C) and resuspended in buffer A (25 mM sodium phosphate buffer, 300 mM NaCl and 25 mM imidazole, pH 8.0) supplemented with DNase I, lysozyme and complete EDTA-free protease inhibitor cocktail tablets (Roche). Cells were lysed at 30 kPsi using a continuous flow cell disruptor (Constant systems) and soluble proteins obtained by centrifugation (20 000×***g***, 30 min and 4°C). The soluble fraction was loaded onto a 5-ml HisTrap HP column and the bound protein eluted with a gradient of imidazole (0–250 mM) in buffer A. Fractions containing the protein of interest were pooled and the hexahistidine tag was removed with TEV protease overnight at 4°C while dialysing against 25 mM sodium phosphate, 300 mM NaCl, pH 8.0, in a 10 000 MWCO membrane. Pure protein was observed after a final HisTrap HP purification step.

### Oligomeric structure

Recombinant proteins were loaded onto a Superdex 75 10/300 or Superose 12 10/300 column, equilibrated in 25 mM HEPES, 150 mM NaCl, pH 7.33 and the elution volume of the recombinant proteins was compared with that of known protein standards (Bio-Rad). *Tc*AKR separations were also repeated using the Superose 12 10/300 column with the high salt buffer (150 mM Tris–HCl, pH 7.6, 300 mM NaCl) as previously described [21].

### Enzyme assays

Enzymatic activity was assessed by measuring the continuous oxidation of NADPH at 340 nm in the presence of varying concentrations of substrate using a Shimadzu UV-2401 or 1601 spectrophotometer. Measurements were taken under steady-state conditions, with a fixed concentration of NADPH (100 µM) in 25 mM HEPES, 150 mM NaCl, pH 7.33, using an appropriate amount of enzyme. Enzyme concentrations for measuring the specific activities were quantified with the Bradford protein assay using bovine serum albumin as standard. Enzyme concentration for kinetic characterisation was determined by UV spectroscopy using the molar extinction coefficients of 37 735 for *Hs*PGFS, 60 470 for *Tc*AKR, 51 590 for *Tb*PGFS, 52 930 for *Li*PGFS1 and 51 530 for *Li*PGFS2 calculated using CLC workbench version 6.9.1. The observed rate of change at 340 nm was converted into s^−1^ prior to fitting the data to the Michaelis–Menten kinetics equation in GraFit 5.0 yielding apparent values for *k*_cat_ and *K*_m_. Reported values for *K*_m_ $\left(K_{m}^{\mathrm{app}}\right)$ and *k*_cat_ values are the weighted mean of independent experiments. Weighted means and standard deviations were calculated using the following equations [23], where *A*, *B*…*I* are the means and *a*, *b*…*i* are standard deviations of *A*, *B*, etc.

Weighted mean of individual mean values

$$=\frac{\left(A/a^{2})+(B/b^{2})+\ldots +(I/i^{2}\right)}{\left(1/a^{2})+(1/b^{2})+\ldots +(1/i^{2}\right)}$$

Weighted mean of standard deviations

$$=\frac{\left(1/a)+(1/b)+\ldots +(1/i\right)}{\left(1/a^{2})+(1/b^{2})+\ldots +(1/i^{2}\right)}$$

### Recombinant prostaglandin F_2α_ synthase assay

A total of 1 mg of purified proteins (*Tc*AKR, *Li*PGFS1, *Li*PGFS2, *Tb*PGFS, *Hs*PGFS and *Tc*OYE) were incubated in the enzyme assay buffer in the presence of 10 µM prostaglandin H2 (Cayman Chemicals) and 100 µM NADPH for 1 h at 37°C. Samples were inactivated by the addition of two volumes of acetonitrile and analysed by LC–MS/MS as described below for the analysis of arachidonic acid and a prostaglandin standard mixture (Cayman Chemicals).

### UPLC-QToF/MS analysis of benznidazole metabolism

Recombinant *Tc*AKR (13 µg and 130 µg ml^−1^) was incubated with 100 µM benznidazole and 100 µM NADPH for 1 h at 37°C in 25 mM HEPES, 150 mM NaCl, pH 7.33. Hypotonic lysates (40 mg) made from *T. cruzi* wild-type or NTR1-overexpressing epimastigotes were incubated with 100 µM benznidazole and 1 mM NADH as described above as a positive control. Reactions with pure enzyme or crude parasite lysates were terminated by the addition of two volumes of acetonitrile and analysed by UPLC-QToF/MS. Metabolite identification samples were analysed on a Waters Acquity UPLC coupled to a Waters Xevo QToF mass spectrometer. Chromatographic separation was achieved on a Waters BEH C18 column (50 × 2.1 mm, 1.7 µm particle size) eluted with A: water + 0.1% formic acid, B: acetonitrile + 0.1% formic acid. A flow rate of 0.5 ml min^−1^ and an injection volume of 5 µl were selected. Analysis was performed with a 7 min LC run: 98% A initially, to 65% A after 4 min then 5% A after 5 min, held for 0.99 min before returning to 98% A after 6 min. MS^E^ spectra were acquired, allowing exact mass precursor and fragment data to be simultaneously collected over a mass range of 50–1200 *m*/*z* with the following mass spectrometer parameters: positive electrospray ionisation, cone voltage 38 V, mass range 100–1200 *m*/*z*, ramping collision energy 20–40 V, capillary voltage 1.5 kV, desolvation temperature 500°C, source temperature 120°C, desolvation gas (nitrogen) 800 l h^−1^ and cone gas (nitrogen) 10 l h^−1^.

### Parasite growth and electroporation

Epimastigotes of the *T. cruzi* strain Silvio X10/7 (MHOM/BR/78/Silvio; clone X10/7) clone A1 were grown RTH/FCS medium at 28°C [24]. *T. brucei* bloodstream-form parasites were grown in an HMI9-T [25] medium at 37°C and *Leishmania donovani* (MHOM/SD/62/1S-CL2D) promastigotes were grown in a modified M199 medium described previously [26]. Transgenic parasites overexpressing NTR1 (TCSYLVIO_001958) were generated by transfection of plasmid pTREX-TcNTR1 with an Amaxa Nucleofector 2b device using program U-33. Transgenic parasites were selected by the addition of G418 at 250 µg ml^−1^.

### Arachidonic acid co-culture experiment

*T. brucei* bloodstream-form parasites, *Leishmania donovani* promastigotes and *T. cruzi* epimastigotes, were incubated in the presence of 66 µM arachidonic acid (Sigma–Aldrich) for 16 h at 37°C, 28°C and 28°C, respectively. Parasites were collected by centrifugation and washed in PBS three times prior to biological inactivation by three rounds of freeze–thaw before mixing with two volumes of acetonitrile and analysis by LC–MS/MS.

### Lysate arachidonic acid experiment

*T. cruzi* epimastigotes and *T. brucei* BSF were harvested by centrifugation at 800×***g*** and washed three times in ice cold PBS. Parasites (1 × 10^8^) were collected and lysed by hypotonic lysis (10 mM Tris–HCl, pH 7.5) for 5 min on ice. Incubations with and without 1 mM arachidonic acid were carried as previously described [19], in a reaction volume of 400 µl and reactions terminated by the addition of 800 µl acetonitrile. Samples were subjected to three rounds of freeze–thaw treatment to ensure biological inactivation, prior to analysis by LC–MS/MS. All samples were analysed on a Waters TQ-S with Acquity UPLC using a Waters BEH C18 column (50 × 2 mm, 1.7 µm particle size). Source temperature was 150°C and desolvation gas temperature was 600°C. Eluents used were A: water + 0.01% formic acid, B: acetonitrile + 0.01% formic acid and the flow rate was 0.5 ml min^−1^. Initial work on the incubation of arachidonic acid with parasites was performed with a 6 min LC run: 95% A initially, to 80% A after 1 min then 60% A after 4 min, to 5% A after 4.5 min, held for 0.5 min before returning to 95% A after 5.1 min. Further work on the incubation of arachidonic acid with cell lysates was performed with a 10 min LC run: 95% A initially, to 80% A after 1 min then 60% A after 8 min, to 5% A after 8.1 min, held for 0.9 min before returning to 95% A after 9.1 min. Multiple reaction monitoring (MRM) transitions used for the prostaglandin species monitored are listed in Table 2 and Supplementary Figure S1 (all in negative ion mode).

**Table 2:** MRM transitions used for detecting the prostaglandin species and their retention times Chromatograms of the 10 min chromatographic separations are provided in Supplementary Figure S1.

Prostaglandin	MRM transition	Retention time (min)
6-keto PGF_1α_	369.2 > 163.1	3.09
Iso-PGF_2α_	353.0 > 192.9	4.25
PGF_2α_	353.0 > 192.9	4.88
PGF_1α_	355.2 > 311.2	4.96
PGE_2_	351.1 > 271.1	5.06
PGE_1_	353.2 > 317.2	5.31

## Results

### Characterisation of TcAKR

**Figure 1.**
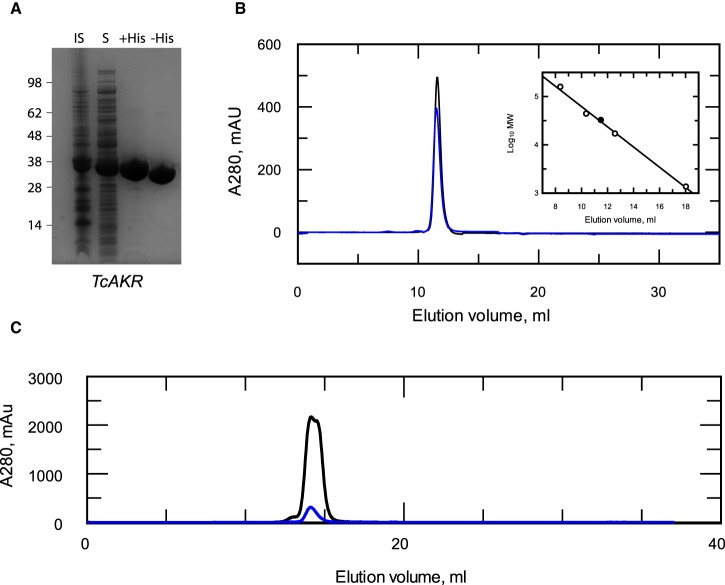
Characterisation of recombinant *Tc*AKR. (**A**) Purity of recombinant enzyme by SDS–PAGE: insoluble (lane 1), soluble (lane 2), pooled fraction after HisTrap (lane 3) and pooled protein after hexahistidine tag removal (lane 4). (**B**) Elution profile of recombinant *Tc*AKR (black) and His_6_-*Tc*AKR (blue) under quasi-physiological conditions from a Superdex 75 10/300 column. (Inset) Elution volumes of known protein standards (open circles) plotted against the log_10_ of their molecular masses. Theoretical elution volume of the *T. cruzi* AKR monomer (closed circle). Linear fit *R*^2^: 0.998. (**C**) Elution profile of recombinant *Tc*AKR at 2 mg ml^−1^ (black) and 20 mg ml^−1^ (blue) under quasi-physiological conditions from a Superose12 10/300 column.

The enzymatic function of *Tc*AKR is far from clear: it is annotated as a prostaglandin F synthase in some genomic databases, yet the recombinant protein is reported to lack such activity [21] and other possible physiological substrates have not been tested. It has also been proposed that *Tc*AKR plays a role in the metabolism of, and resistance to, benznidazole [15,16]. To clarify these issues, *Tc*AKR was expressed in *Escherichia coli*, purified to homogeneity (Figure 1A) and its physico-chemical properties were determined. The purified protein (with or without the His_6_-tag) was analysed by size exclusion chromatography and found to elute as a single symmetrical peak with a calculated *M*_r_ close to that of a theoretical monomer (Figure 1B), when separated under quasi-physiological conditions (pH 7.33, 150 mM NaCl). This finding is in complete contrast with that of a previous study by Garavaglia et al. [21], who reported the presence of mixed species containing monomeric, dimeric and tetrameric forms of the enzyme from the CL-Brener strain of *T. cruzi*. When repeating these authors’ conditions using the same column and buffer conditions, the *Tc*AKR isolated from our laboratory strain of *T. cruzi* continued to elute as a monomeric species, irrespective of the amount loaded (Figure 1C). The reason for this discrepancy is not clear. Both enzymes are highly similar (97% amino acid sequence identity); however, modelling onto the published crystal structure of this enzyme [27] suggests that the majority of these differences are located on the surface. Notably, one allele in the CL-Brener strain contains an additional cysteine (Y51C) (SNP: NGS_SNP.TcChr40-P.486408), which is absent in the Silvio X10-7 clone used in our study. Modelling on the *Tc*AKR crystal structure 4GIE [27] suggests that this cysteine residue is likely to be solvent accessible and therefore available for disulfide formation with another monomer. This could account for dimer (but not the tetramer) species reported by Garavaglia et al. [21] and would explain why their dimeric species did not dissociate into monomers during mass spectrometry.

### Substrate-specificity of TcAKR

**Figure 2.**
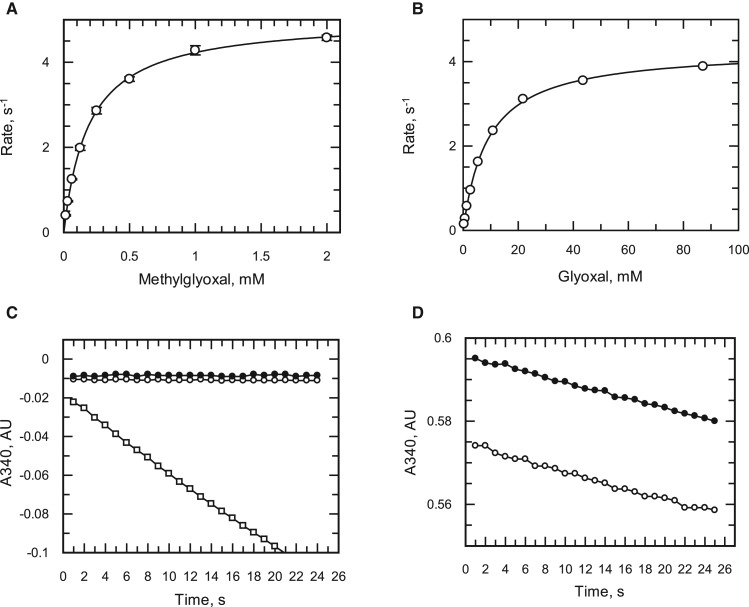
Testing benznidazole as a substrate or inhibitor of recombinant *Tc*AKR. (**A**) Benznidazole as a substrate of TcAKR, DMSO (open circles), 60 µM benznidazole (closed circles), 60 µM phenylglyoxal (open squares). (**B**) Inhibition of phenylglyoxal (60 µM) metabolism by benznidazole, DMSO (closed circles), 60 µM benznidazole (open circles). (**C** and **D**) Michaelis–Menten plots of activity with glyoxal and methylglyoxal, respectively.

As *Tc*AKR has no clear biological role, a BLAST analysis was carried out in an attempt to assign a possible function for this enzyme, identifying enzymes in vitamin K biosynthesis, glyoxal metabolism and synthesis of prostaglandin F_2α_. The activity of *Tc*AKR was assessed with a fixed concentration of a variety of biologically relevant or typical AKR substrates (Table 3) identifying methylglyoxal, a toxic by-product of glycolysis, to have one of the highest specific activities with the purified enzyme. Subsequent characterisation of glyoxal and methylglyoxal showed them to follow Michaelis–Menten kinetics (Figure 2A,B). Notably, methylglyoxal had a *K*_m_ of 0.24 mM and *k*_cat_/*K*_m_ (1.95 × 10^4^ M s^−1^) which is similar to that of SakRI (1.1 × 10^4^ M^−1^ s^−1^), a cyanobacterial enzyme involved in methylglyoxal detoxification [18].

**Table 3:** A survey of purified recombinant trypanosomatid aldo-keto reductases with various substrates Compounds (60 µM) were assayed spectrophotometrically in the presence of 100 µM NADPH. Specific activity is given in µmol min^−1^ mg^−1^. Values are the means and standard deviations (*n* = 3).

Substrate	*Tc*AKR	*Li*PGFS1	*Li*PGFS2	*Tb*PGFS
2-Nitrobenzaldehyde	0.20 ± 0.004	1.40 ± 0.04	2.51 ± 0.029	0.73 ± 0.017
3-Nitrobenzaldehyde	0.16 ± 0.003	0.06 ± 0.002	2.40 ± 0.037	1.86 ± 0.009
4-Nitrobenzaldehyde	0.39 ± 0.009	0.06 ± 0.002	2.49 ± 0.142	0.71 ± 0.013
Glyoxal	0.001 ± 0.001	0.02 ± 0.001	0.09 ± 0.001	0.15 ± 0.005
Methylglyoxal	1.20 ± 0.022	0.61 ± 0.02	1.79 ± 0.009	0.67 ± 0.012
Phenylglyoxal	3.66 ± 0.092	3.11 ± 0.018	2.46 ± 0.084	0.78 ± 0.037
Vitamin K3 epoxide	N.A.	N.A.	N.D.	N.A.
Glyceraldehyde	0.05 ± 0.005	N.D.	0.16 ± 0.002	0.13 ± 0.004
Benznidazole	N.A.	N.A.	N.A.	N.A.

Abbreviations: NA, not active; ND, not determined.

**Figure 3.**
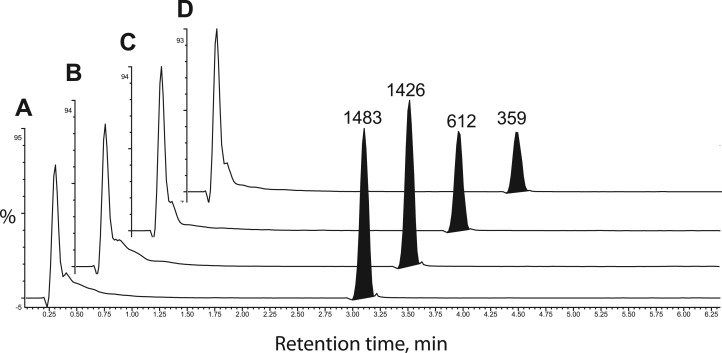
Metabolism of benznidazole by TcAKR and whole cell lysates of *T. cruzi.* Extracted ion chromatograms measuring the relative amount of benznidazole in the presence of NADH plus (**A**) no enzyme, (**B**) 0.13 mg ml^−1^ recombinant *Tc*AKR, (**C**) 40 mg ml^−1^ of a wild-type *T. cruzi* lysate and (**D**) 40 mg ml^−1^ of a lysate-overexpressing NTR.

Although benznidazole was recently reported to be a substrate of this enzyme (73 mU mg^−1^) [16], we were unable to detect any increase in NADPH oxidation above the background rate (1 mU mg^−1^) when benznidazole was present at 60 µM (Figure 2C). Neither did we observe any inhibition of enzymatic activity by benznidazole with phenylglyoxal as a substrate (Figure 2D). To confirm our negative findings with the spectrophotometric assay, the purified enzyme was incubated with NADPH in the presence or absence of benznidazole for 1 h at 37°C and subsequently analysed by UPLC-QToF/MS. Even in the presence of excess enzyme (130 µg ml^−1^), we were unable to detect any utilisation of benznidazole (Figure 3A,B). In contrast, incubation of benznidazole with parasite lysates under identical conditions converted more than half of the benznidazole (Figure 3C,D) into multiple metabolites already reported in the literature (Table 4) [8,10]. The smaller peak size of benznidazole incubated with the lysate prepared from NTR-overexpressing parasites (Figure 3D) is consistent with NTR-activating benznidazole [8]. Other clinically pertinent nitro-drugs for the trypanosomatid parasites, nifurtimox, fexinidazole [5] and (*R*)-PA824 [28] were also not substrates for AKR. These results demonstrate that *Tc*AKR is not directly involved with the bio-activation of benznidazole or other nitro-drugs.

**Table 4:** Benznidazole-derived metabolites Metabolites were identified by accurate mass and presence in the sample, but absence in the control incubations.

Retention time (min)	Structure	Metabolite number^1^	[M+H]^+^	Ionisation state	WT lysate	NTR^OE^ Lys B	Control	TcAKR
3.1	Benznidazole	NA	261.10	1	NA	NA	NA	NA
1.59	Hydroxylamine or hydroxy derivative 1 or 2	11 or 19	247.11	1	Y	Y	N	N
1.8	Amino derivative of benznidazole	1	231.12	1	Y	Y	N	N
1.49/1.59	Dihydroxy-dihydro derivative	3	265.13	1	Y	Y	N	N
1.59	*N*-benzyl-2-guanidinoacetamide	5	207.13	1	N	Y	N	N
1.3	2-Amino-*N*-benzylacetamide	22	165.10	1	Y	Y	N	N
1.74	Amino derivative + glutathione (isomer 1)	7 or 8	536.19	1	Y	Y	N	N
			268.56	2	Y	Y	N	N
1.83	Amino derivative + glutathione (isomer 2)	7 or 8	536.19	1	Y	Y	N	N
			268.56	2	Y	Y	N	N
1.55/1.64	Amino derivative + glutathione + H_2_O	4	554.20	1	Y	Y	N	N
			277.60	2	Y	Y	N	N
1.76/1.93/2.31	Amino derivative + cysteine	9	350.13	1	Y	Y	NN	NN
1.34	Amino derivative + ovothiol A	12 or 14	430.17	1	Y	Y	N	N
			215.58	2	Y	Y	N	N
3.8	Possible benznidazole adduct	26	385.18	1	Y	Y	N	N
1.63	Possible benznidazole adduct	28	368.12	1	N	Y	N	N
3.64/3.8	Possible benznidazole adduct	NA	614.35	1	Y	Y	N	N
2.13	Possible benznidazole metabolite	NA	204.05	1	Y	Y	N	N

Abbreviations: NA, not applicable.

1 The metabolite numbers refer to the metabolite list in Trochine et al. [10].

### Identifying alternative methylglyoxal-reducing enzymes in the trypanosomatids

**Figure 4.**
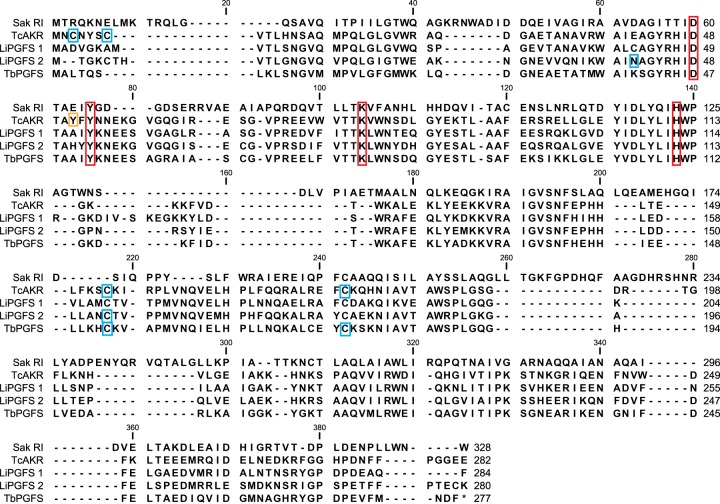
Sequence alignment of AKR homologues identified by BLAST. Multiple sequence alignment of SakRI, *Tc*AKR (TcCLB.511287.49), *Li*PGFS1 (LinJ.31.2210), *Li*PGFS2 (LinJ.32.0470) and *Tb*PGFS (Tb927.11.4700). The conserved AKR catalytic tetrad identified in *T. brucei* is marked by the red boxes. Blue boxes indicate solvent accessible cysteine residues identified in the crystal structures of *Tb*PGFS *Tc*AKR and *Li*PGFS2 (PDB accession numbers 1VBJ, 4GIE and 4G5D) [27]. Orange box highlighting Y51C allelic variation was observed in CL-Brener.

**Figure 5.**
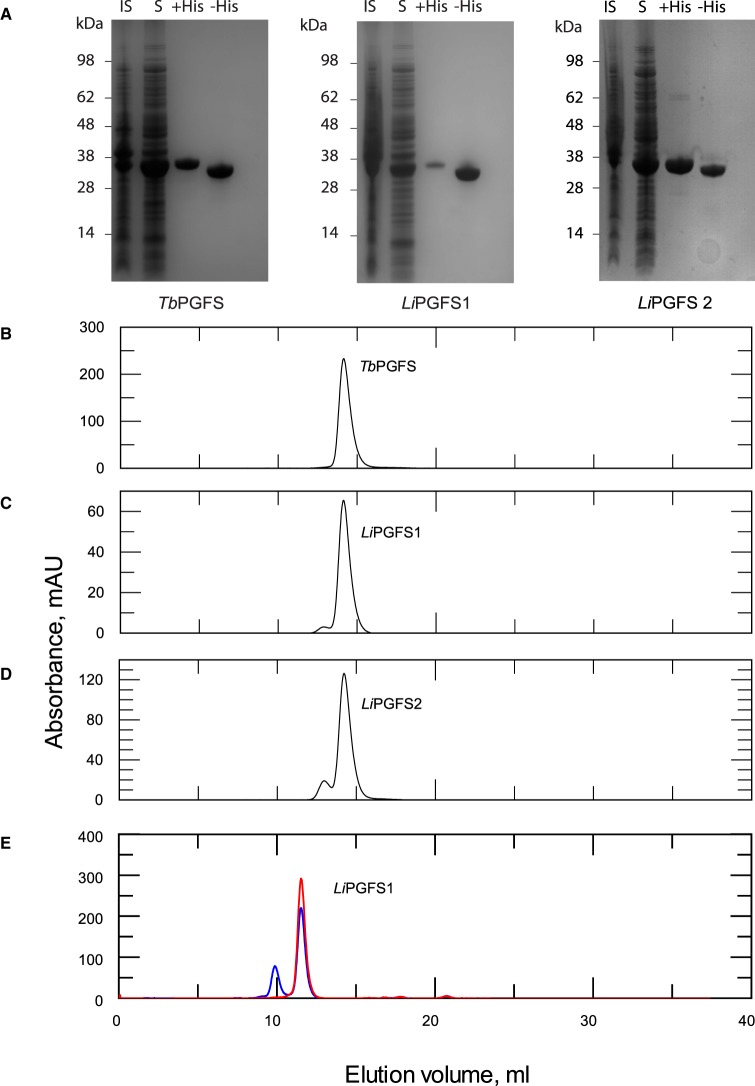
Characterisation of *Li*PGFS1, *Li*PGFS2 and *Tb*PGFS. (**A**) SDS–PAGE showing purification of *Tb*PGFS1, *Li*PGFS1 and *Li*PGFS2 showing insoluble, soluble, pooled protein from HisTrap column and protein with the hexahistidine tag removed. Size exclusion elution profiles of (**B**) *Tb*PGFS, (**C**) *Li*PGFS1 and (**D**) *Li*PGFS2. Samples were separated on a Superose 12 10/300 column in 25 mM HEPES, 150 mM NaCl, pH 7.33. (**E**) LiPGFS1 stored in the absence of a reducing agent analysed by size exclusion chromatography in the absence (blue) and presence (red) of 0.5 mM TCEP using a Superdex 75 10/300 column.

Homologues of *Tc*AKR were identified in the related parasites *T. brucei* and *L. infantum*, identifying one and two homologues, respectively. The identification of a *T. brucei* homologue was of particular interest as this parasite notably lacks the trypanothione-dependent glyoxalase pathway present in *Leishmania* and *T. cruzi* [29]. The genes encoding these enzymes have been annotated as prostaglandin F synthases, with the *L. infantum* (LinJ.31.2210) and *T. brucei* (Tb927.11.4700) sequences previously assigned to AKR sub-family 5A [17]. Alignments revealed the trypanosomatid sequences to share between 54 and 57% identity with each other (Figure 4) with all maintaining the conserved AKR catalytic tetrad identified in *T. brucei* [30]. To further characterise the enzymatic properties of these proteins, the homologues identified from *T. brucei* and *L. infantum* were overexpressed in *E. coli* and purified to homogeneity using the same two-stage purification as for *Tc*AKR (Figure 5A). The typical yields of pure protein ranged between 10 and 30 mg l^−1^ of starting culture. Similar to *Tc*AKR, the homologues *Tb*PGFS, *Li*PGFS1 and *Li*PGFS2 were primarily monomeric (Figure 5B–D) when analysed by size exclusion chromatography under the same quasi-physiological conditions. However, when *Li*PGFS1 was incubated for 96 h at 4°C in the absence of a reducing agent, 25% of the protein eluted with an apparent mass of a dimer (Figure 5E). The addition of a reducing agent completely abolished the dimeric species (Figure 5E), indicating the dimeric form to be reliant upon disulfide bond formation.

### Comparative kinetics of trypanosomatid AKRs

The enzymatic properties of the three additional purified homologues were assessed with the same substrates tested against *TcAKR*, finding that all of the trypanosomatid enzymes were capable of using the same substrates (Table 3). Similar to *Tc*AKR, neither the *Leishmania* nor *T. brucei* enzymes were capable of using the nitro-drugs benznidazole or nifurtimox as substrates. As the homologues displayed identical substrate usage, the basic kinetic parameters of each enzyme for each of the substrates were determined at a fixed concentration of NADPH (Table 5). Comparing the apparent $K_{m}^{\mathrm{app}}$ values, substrate turnover rates and catalytic efficiencies of the enzymes revealed *Li*PGFS1 to be more kinetically similar to the *Tc*AKR than *Tb*PGFS, with the opposite relationship true of *Li*PGFS2 suggesting a divergence in AKR evolution in *Leishmania*. We observed the catalytic efficiency (*k*_cat_/*K*_m_) of methylglyoxal as a substrate of these enzymes to be relatively unchanged despite up to 20- and 80-fold differences *k*_cat_ and *K*_m_ values, respectively. This trend also was observed for glyoxal, phenylglyoxal and d,l-glyceraldehyde. However, the nitro-aromatic compounds were much more efficiently utilised in the case of *Tb*PGFS or *Li*PGFS2 compared with *Li*PGFS1 and *Tc*AKR. *Li*PGFS2 used in our study appears to be the same as a glutathione-specific aldose reductase [31]; however, it appears that glutathione is not required for the functional activity of this enzyme in our hands. The human prostaglandin F_2α_ synthase (NP_003730.4) also displayed trace activity with methylglyoxal as a substrate, but with a much lower efficiency (∼10 M^−1^ s^−1^), indicating that it is unlikely to be a functional mechanism for detoxification of methylglyoxal in human cells.

**Table 5:** Kinetic characterisation of trypanosomatid recombinant aldo-keto reductases with various substrates

Substrate	Units	*Tc*AKR	*Li*PGFS1	*Li*PGFS2	*Tb*PGFS
Glyoxal					
* k*_cat_	s^−1^	4.56 ± 0.05	7.31 ± 0.19	1.05 ± 0.02	ND
* *$K_{m}^{\mathrm{app}}$^1^	mM	8.30 ± 0.31	36.1 ± 2.39	0.56 ± 0.05	ND
* *$k_{\mathrm{cat}}/K_{m}^{\mathrm{app}}$	M^−1^ s^−1^	549	202	1880	ND
Methylglyoxal					
* k*_cat_	s^−1^	4.69 ± 0.03	8.72 ± 0.17	1.13 ± <0.01	0.38 ± 0.007
* *$K_{m}^{\mathrm{app}}$	mM	0.24 ± <0.001	0.80 ± 0.07	0.02 ± <0.001	0.01 ± <0.001
* *$k_{\mathrm{cat}}/K_{m}^{\mathrm{app}}$	M^−1^ s^−1^	19 500	10 900	56 500	38 000
Phenylglyoxal					
* k*_cat_	s^−1^	4.46 ± 0.08	10.4 ± 0.22	1.17 ± 0.02	0.34 ± 0.014
* *$K_{m}^{\mathrm{app}}$	mM	0.05 ± 0.003	0.14 ± 0.008	0.01 ± 0.0009	0.005 ± 0.0008
* *$k_{\mathrm{cat}}/K_{m}^{\mathrm{app}}$	M^−1^ s^−1^	89 200	74 300	58 500	68 000
2-Nitrobenzaldehyde					
* k*_cat_	s^−1^	2.25 ± 0.11^2^	8.50 ± 0.21	1.04 ± 0.02	0.41 ± 0.002
* *$K_{m}^{\mathrm{app}}$	mM	0.71 ± 0.08^2^	0.32 ± 0.02	0.0024 ± 0.0003	0.003 ± 0.0003
* *$k_{\mathrm{cat}}/K_{m}^{\mathrm{app}}$	M^−1^ s^−1^	3170	26 600	433 000	140 000
3-Nitrobenzaldehyde					
* k*_cat_	s^−1^	4.07 ± 0.17	1.28 ± 0.03	1.04 ± 0.02	0.34 ± 0.005
* *$K_{m}^{\mathrm{app}}$	mM	2.20 ± 0.16	1.55 ± 0.06	0.002 ± 0.0003	0.002 ± 0.0001
* *$k_{\mathrm{cat}}/K_{m}^{\mathrm{app}}$	M^−1^ s^−1^	1850	830	520 000	170 000
4-Nitrobenzaldehyde					
* k*_cat_	s^−1^	3.7 ± 0.15	0.48 ± 0.01	1.13 ± 0.05^2^	0.43 ± 0.008
* *$K_{m}^{\mathrm{app}}$	mM	68 ± 0.07	0.43 ± 0.026	0.004 ± 0.0006^2^	0.0043 ± 0.0002
* *$k_{\mathrm{cat}}/K_{m}^{\mathrm{app}}$	M^−1^ s^−1^	5440	1 110	283 000	100 000
d,l-glyceraldehyde					
* k*_cat_	s^−1^	4.94 ± 0.11	8.72 ± 0.11^2^	2.46 ± 0.13^2^	0.37 ± 0.009
* *$K_{m}^{\mathrm{app}}$	mM	1.50 ± 0.17	2.87 ± 0.08^2^	1.07 ± 0.15^2^	0.07 ± 0.005
* *$k_{\mathrm{cat}}/K_{m}^{\mathrm{app}}$	M^−1^ s^−1^	3290	3040	2300	5290

Abbreviations: ND, not determined.

1 $K_{m}^{\mathrm{app}}$ values determined with 100 µM NADPH.

2 Weighted mean of two individual replicates, all others are *n* ≥ 3.

### Arachidonic acid and prostaglandins

Previous studies on these enzymes have suggested that the *T. brucei* and *Leishmania* spp. AKRs function as prostaglandin F_2α_ synthases in these parasites, converting prostaglandin H_2_ (PGH_2_) to the F_2α_ form. While our observations for prostaglandin F_2α_ synthase activity of *Tb*PGFS and *Li*PGFS2 were in agreement with the literature [19,20], we also observed PGF_2α_ activity for *Li*PGFS1 (Table 6). We also confirm the lack of prostaglandin F_2α_ synthase activity of *Tc*AKR as reported by Garavaglia et al. [21].

**Table 6:** Identification of prostaglandin F_2α_ synthase activity in trypanosomatid aldo-keto reductases and old yellow enzyme

Enzyme	Peak area			
	PGF_2α_	PGF_1α_	PGE_2_	PGE_1_
No enzyme control	3185		176 250	984
*Hs*PGFS	6415		187 319	537
*Tc*AKR	4231		190 097	1161
*Li*PGFS1	33 807	536	156 739	393
*Li*PGFS2	38 285	467	149 112	273
*Tb*PGFS	28 568	306	139 483	606
*Tc*OYE	25 216	510	133 298	353

Enzymes were incubated with PGH_2_ as described in Experimental Procedures and analysed by LC–MS. 6-Keto-PGF_2α_ and iso-PGF_2α_ were not detected. PGH_2_ is unstable and in the absence of enzyme is spontaneously rearranged to PGE_2_ [32,33].

**Figure 6.**
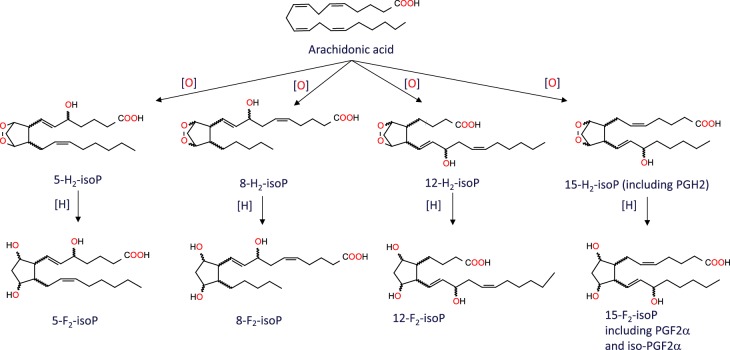
Formation of F_2_-isoprostanes from arachidonic acid. Abbreviations: IsoP, isoprostane.

**Figure 7.**
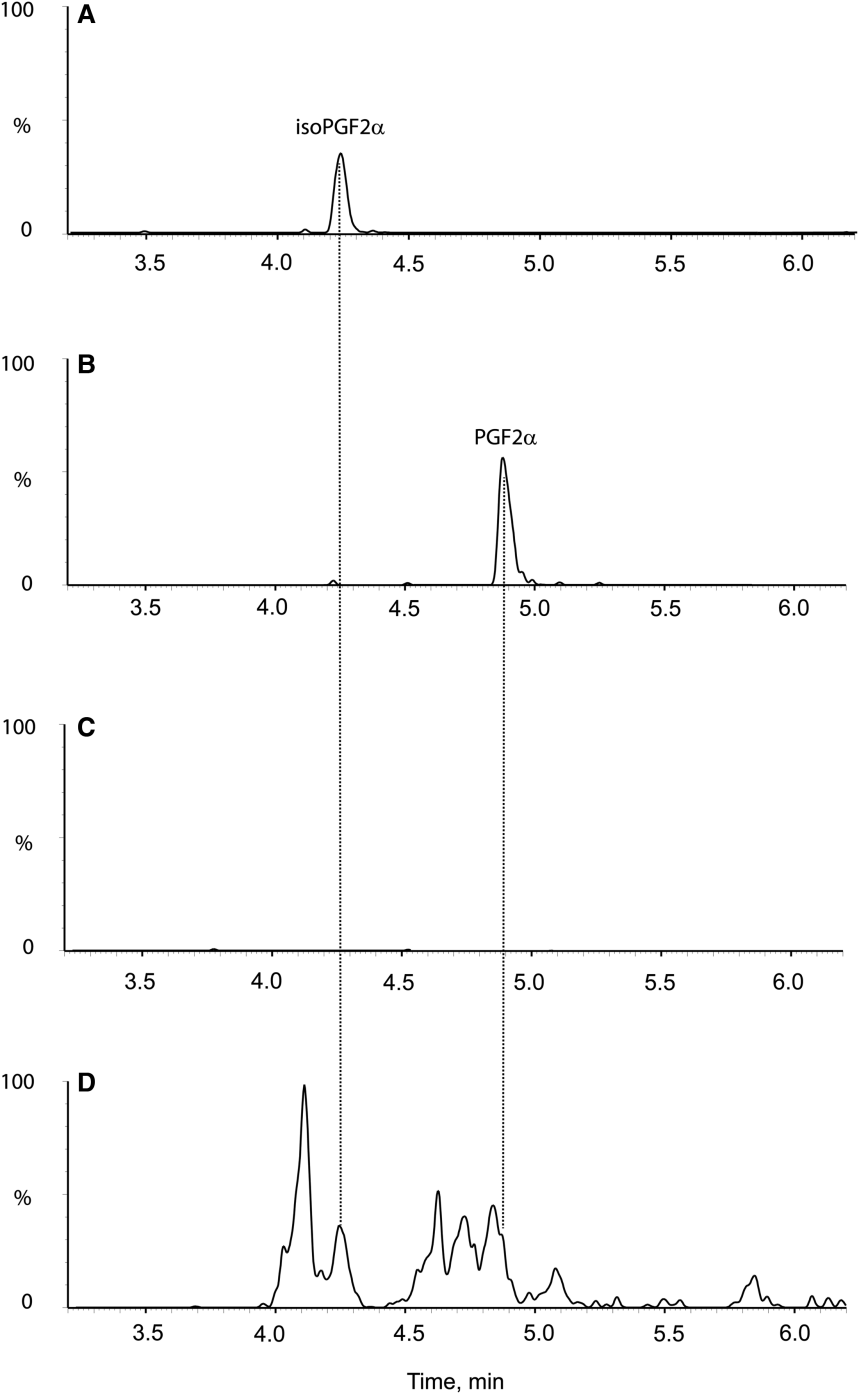
MRM chromatograms monitoring for PGF2α /Iso-PGF2α in *T. cruzi* cell lysates. Elution profile of 2 ng ml^−1^ standards of iso-PGF2α (**A**) and PDF2α (**B**). *T. cruzi* cell lysates incubated with (**D**) or without (**C**) 1 mM arachidonic acid.

Observations that these parasites are capable of synthesising PGH_2_ from arachidonic acid currently do not fit with the known prostaglandin biosynthetic pathway due to the absence of cyclooxygenases 1 and 2 that catalyse the conversion of arachidonic acid to PGH_2_. Our alternative hypothesis is that the formation of the prostaglandins from arachidonic acid could be due to the formation of structurally similar isomers, the isoprostanes. These molecules have been reported as a marker of oxidative stress [34] and are formed by the free radical-mediated peroxidation of arachidonic acid within the cell [35]. In contrast with enzyme-mediated peroxidation that typically generates a single enantiomer of a single regioisomer, non-enzymatic peroxidation generates racemic mixtures with multiple regio- and stereo-centres [36,37]. Among other products, non-enzymatic peroxidation of arachidonic acid produces four F_2_-isoprostane regioisomers (Figure 6), each of which comprises eight racemic diastereomers, making 64 in total [38]. Prostaglandin F_2α_ and its isoprostane 8-isoprostane F_2α_ (15-F2t-IsoP or iPF_2α_-III) belong to the 15-series regioisomers, which, together with the 5-series, are formed in greater amounts than the less stable 8- and 12-series [38]. Although the four F_2_-isoprostane classes are isobaric (*m*/*z* 353), they can be distinguished by their MRM spectra which yield different fragmentation patterns below *m*/*z* 200, with the 15-series (iPF_2α_-III series) producing a predominant 193 anion [39]. The 5-series produces a minor peak at 193, but can be distinguished by its predominant MRM product at *m*/*z* 115 and different retention time on HPLC. To determine if prostaglandins or isoprostanes could be produced, parasites were co-incubated with arachidonic acid and analysed by LC–MS/MS and compared with known analytical standards. Despite the high sensitivity of the LC–MS/MS assay (LOD of ∼0.1 ng ml^−1^ for both species) our initial experiments, where 66 µM arachidonic acid was co-incubated with intact parasites, failed to detect either prostaglandin F_2α_ or its isoprostane 8-isoprostane F_2α_. However, the addition of 1 mM arachidonic acid to hypotonic lysates of *T. brucei* or *T. cruzi* led to the production of multiple isomeric peaks on the MRM channel for PGF_2α_ and iso-PGF_2α_ (Figure 7). A clearly resolved peak was detected for iso-PGF_2α_, along with a shoulder with the same retention time as PGF_2α_. The identity of the other peaks is not known, but is likely to include the other racemic diastereoisomers in the 15-series (Figure 6). Similar results were obtained for both *T. brucei* and *T. cruzi*.

## Discussion

The primary aim of the present study was to assess the capability of *Tc*AKR to reduce or bio-activate clinically relevant monocyclic and bicyclic nitro-drugs that are either in clinical use or under development for the treatment of Chagas disease. Garavaglia et al. [16] reported that recombinant *Tc*AKR and native *Tc*AKR purified from *T. cruzi* epimastigotes were able to reduce benznidazole with specific activities of 0.091 and 0.073 U mg^−1^, respectively. In complete contrast, we found no evidence for the reduction of benznidazole by *Tc*AKR (<0.001 U mg^−1^) (Table 3); neither could we detect metabolism of benznidazole by *Tc*AKR using UPLC-QToF/MS under conditions where extensive metabolism occurred in whole cell lysates (Figure 3 and Table 4). It is unlikely that amino acid substitutions in these AKRs are responsible for the discrepancy, because the CL-Brener and Silvio enzymes are 97% identical and molecular modelling shows that the active sites are identical. Differences in assay conditions could be a possible explanation: our assays were at pH 7.33 in HEPES buffer, whereas Garavaglia et al. used Tris–HCl, pH 6.5—an inappropriate buffer system since the p*K*_a_ for Tris is 8.3. Notably, these authors reported difficulties in measuring catalytic activity because of non-enzymatic reaction between benznidazole and NADPH cofactor under their experimental conditions. We did not encounter any such problem when pure recombinant *Tc*AKR was assayed under our conditions by spectrophotometry (Figure 2) or by mass spectrometry (Table 4). We conclude that *Tc*AKR is not involved in the bioactivation of benznidazole, but instead we offer a potential role for TcAKR in resistance to benznidazole as discussed below.

A second aim of our study was to elucidate the possible metabolic functions of these AKR homologues across the Trypanosomatidae. All four enzymes across three representative species possess activity with ketoaldehydes with specificity constants (*k*_cat_/*K*_m_) in the order phenylglyoxal > methylglyoxal > glyoxal. Notably, all four enzymes had specificity constants for methylglyoxal (range 1.1–5.7 [×10^4^] M s^−1^) that are similar to that of SakRI (1.1 × 10^4^ M^−1^ s^−1^), a bacterial enzyme that has been implicated in methylglyoxal metabolism [18]. The specificity constant for *Tb*PGFS with methylglyoxal (3.8 × 10^4^ M^−1^ s^−1^) is an order of magnitude lower than that reported with PGH_2_ (7 × 10^5^ M^−1^ s^−1^) [30], whereas the reverse is observed for *Li*PGFS2 with methylglyoxal (5.7 × 10^4^ M^−1^ s^−1^) preferred over PGH_2_ (8.8 × 10^3^ M^−1^ s^−1^) [20]. As previously reported [21] and confirmed here, the *T. cruzi* enzyme lacks prostaglandin F synthase activity; this is provided instead by old yellow enzyme [32], a protein that is absent in *T. brucei* and *L. infantum*. Given the broad spectrum of activity with ketoaldehydes, aromatic aldehydes, erythroses (the present study) and quinones [16,19,40], these enzymes are more like carbonyl reductases (EC 1.11.184) [41] than prostaglandin F synthases (EC 1.1.1.188). Of the physiologically relevant substrates examined here, methylglyoxal is a more efficient substrate for all of these enzymes compared with d,l-glyceraldehyde or glyoxal. Enzymes typically operate in cells at substrate concentrations near to or within 10-fold below their *K*_m_ [42,43]. Although the concentration of methylglyoxal is not known for these parasites, the $K_{m}^{\mathrm{app}}$ values (0.01–0.8 mM) for methylglyoxal determined here are consistent with physiological levels of free metabolite of <5 µM reported in other cells [44]. The *K*_m_ values are thus consistent with a role in defence against carbonyl stress and are at the lower range of *K*_m_ values reported for broad specificity methylglyoxal reductases from *Aspergillus* (1.43 and 15.4 mM) [45], *Saccharomyces* (5.9 mM) [46], *Kluyveromyces* (0.43 and 1.6 mM) [47] and *Synechococcus* sp. (0.08 mM) [18].

Our results suggest an important biological role in the detoxification of toxic ketoaldehydes typified by methylglyoxal, formed as a by-product of the glycolytic pathway, as well as from threonine degradation and from lipid peroxidation [48,49]. Our previous studies have identified two pathways for degradation of methylglyoxal in trypanosomatids, either by conversion to d-lactate catalysed by trypanothione-dependent glyoxalases GLO1 and GLO2, or by conversion to l-lactate via methylglyoxal reductase and lactaldehyde dehydrogenase [25]. Methylglyoxal is formed mainly from triose phosphate intermediates in glycolysis, a pathway whose first nine steps, including isomerisation of dihydroxyacetone phosphate and glyceraldehyde 3-phosphate, take place in a peroxisome-like organelle, the glycosome [50]. It is of interest to note that PGFS and GLO2 are present in highly purified glycosomal preparations from *T. brucei* [51] and, likewise, PGFS, GLO1 and two putative d-lactate dehydrogenases are found in *L. donovani* glycosomes [52], suggesting a role for both pathways in protection against protein glycation [48] in these organelles as well as in the cytosol. Curiously, bloodstream forms of *T. brucei* have the highest glycolytic flux of all these trypanosomatids [53], yet lack GLO1 of the trypanothione-dependent glyoxalase pathway present in *Leishmania* spp. and *T. cruzi* [25].

**Figure 8.**
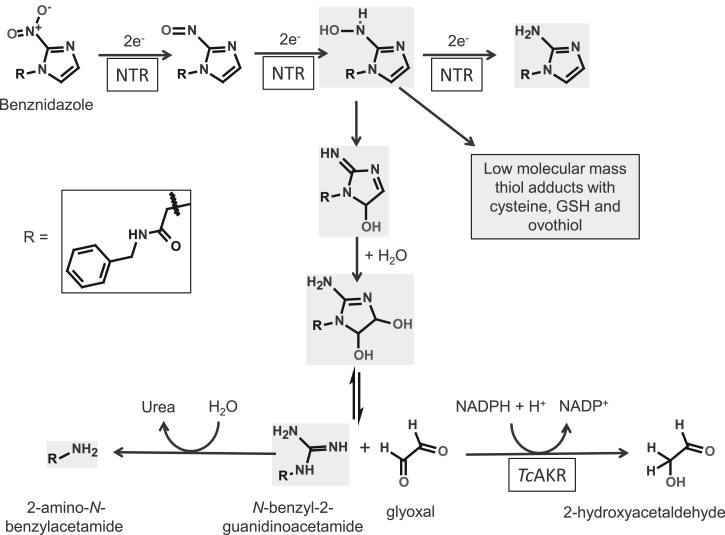
Possible role of AKR in the metabolism of benznidazole. The metabolites shaded in grey were identified in our study (Table 4). The removal of glyoxal by AKR promotes further metabolism to 2-amino-*N*-benzyl acetamide.

In the case of *Tc*AKR, overexpression of this enzyme confers a modest resistance to benznidazole [16]. Benznidazole is a prodrug and undergoes activation via two sequential two-electron reduction steps by a mitochondrial nitroreductase to form a toxic reactive hydroxylamine intermediate (Figure 8). This compound subsequently forms adducts with low molecular mass thiols [10] or undergoes rearrangement to a dihydro-dihydroxy intermediate [8] that can dissociate to form glyoxal and *N*-benzyl-2-guanidinoacetamide [54] (Figure 8). Glyoxal is toxic and mutagenic and reacts with DNA to form a cyclic glyoxal-deoxyguanosine adduct, as well as causing deamination of deoxycytidine and cross-linking in DNA [55]. Thus, overexpression of *Tc*AKR may contribute to benznidazole resistance by facilitating removal of glyoxal.

Given the absence of genetic and biochemical evidence for cyclooxygenase-dependent synthesis of PGH_2_ from arachidonic acid [19,20,32], the evidence for specific prostaglandin metabolism requires critical reassessment. In mammalian tissues, non-cyclooxygenase-derived F_2_-isoprostanes can be formed *in situ* on arachidonic acid-containing phospholipids via lipid peroxidation and then released by the action of phospholipase A_2_ [35]. Alternatively, they can be formed from free arachidonic acid *in vitro* [56]. *Leishmania* spp. are able to synthesise arachidonic acid *de novo* [57], whereas *T. cruzi* and *T. brucei* are able to scavenge it from the extracellular medium and incorporate it into lipid bodies [57–59]. All species contain phospholipase A_2_ [60,61]. Our findings support the non-enzymatic formation of PGH_2_ and other F-type isoprostanes by free radical peroxidation of arachidonic acid. Notably, the formation of multiple isobaric products (*m*/*z* 353) with the characteristic MRM signal for the 15-series of F-type prostanoids (*m*/*z* 193) supports a non-enzymatic peroxidation mechanism, in contrast with a cyclooxygenase-catalysed peroxidation that would generate a single enantiomerically pure product [36,37]. In this respect, it is worth noting that the four studies reporting enzymatic conversion of arachidonic acid into PGD_2_, PGE_2_ and PGF_2α_ utilised an analytical approach that focuses exclusively on these prostanoids [19,32,40,59] and predates the discovery of isoprostanes. The method involves incubation of whole cell lysates with saturating amounts of arachidonic acid, spiking with trace amounts of radiolabelled PGD_2_, PGE_2_ and PGF_2α_, separation by HPLC and quantitation of radiolabelled peaks by enzyme immunoassay [62]. Thus, the large number of additional F_2_-isoprostane metabolites observed here (Figure 7) may have been missed. We propose that arachidonic acid (free or as phospholipid) undergoes non-enzymatic peroxidation to form 8-iso-PGH_2_ (among other enantiomers) which serves as a substrate for AKRs to form 8-iso-PGF_2α_ (and other isoprostanes). Additionally, we note that a report that *T. cruzi* produces thromboxane A_2_ as well as PGF_2α_ [63] could also involve a non-enzymatic pathway [64]. Thus, there is a need for a thorough re-evaluation of the nature of eicosanoids formed in these parasites and the possible physiological role of the products. For example, it is worth noting that host immune responses to parasite infection often involve oxidant stress [65,66] and that certain prostanoids formed via the free radical route can have biological activity [67].

In conclusion, we have demonstrated that the substrate-specificity of these aldo-keto reductases is broader than other prostaglandin F_2α_ synthases and that they are likely to be involved in the elimination of certain toxic ketoaldehyde metabolites derived from lipids, trioses and the drug, benznidazole.

## Abbreviations

AKR: aldo-keto reductase
MRM: multiple reaction monitoring
NTR: nitroreductase
ORFs: open reading frames
PGF2α: prostaglandin F_2α_
PGH_2_: prostaglandin H
