# Supplementary material for: A role for trypanosomatid aldo-keto reductases in methylglyoxal, prostaglandin and isoprostane metabolism
Source: Biochem J. 2018 Aug 29;475(16):2593–610. doi: 10.1042/BCJ20180232 (PMC6117947; doi:10.1042/BCJ20180232)
Supplement: Supplementary Figure S1 [file BCJ-475-2593-s1.pdf]

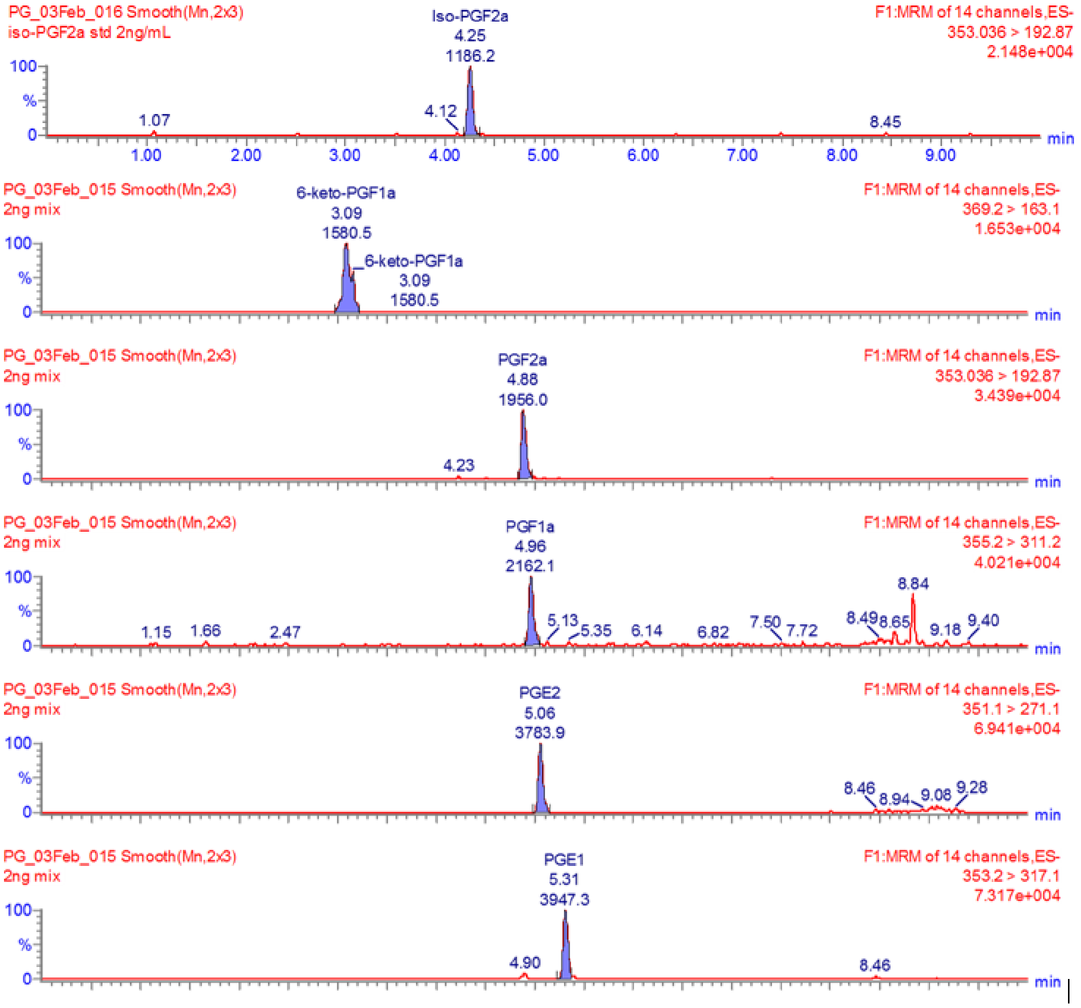

Figure S1
MRM chromatograms of a 2ng ml^{-1} standard of Iso-PGF2α standard (top) and mix of prostaglandin standards at 2ng ml^{-1} (lower 5 traces) showing retention time and peak areas obtained.
